# Radiating pain during epidural needle insertion and catheter placement cannot be associated with postoperative persistent paresthesia: a retrospective review

**DOI:** 10.1186/s40981-021-00460-w

**Published:** 2021-08-19

**Authors:** Taichi Kotani, Satoki Inoue, Keiko Uemura, Masahiko Kawaguchi

**Affiliations:** 1 Department of Anesthesiology and Division of Intensive Care, Nara Medical University, 840 Shijo-cho Kashihara, Nara, 634-8522 Japan; 2 Department of Anesthesiology, Fukushima Medical University, 1 Hikarigaoka, Fukushima, Fukushima 960-1295 Japan

**Keywords:** Epidural anesthesia, Radiating pain, Paresthesia

## Abstract

**Background:**

It has been suggested that radiating pain during spinal or epidural needle insertion and catheter placement can be an indicator of needle-related nerve injury. In this study, using a historical cohort, we investigated what factors could be associated with postoperative persistent paresthesia. In addition, we focused on radiating pain during epidural needle insertion and catheterization.

**Methods:**

This was a retrospective review of an institutional registry containing 21,606 anesthesia cases. We conducted multivariate logistic analysis in 2736 patients, who underwent epidural anesthesia, using the incidence of postoperative persistent paresthesia as a dependent variable and other covariates, including items of the anesthesia registry and the postoperative questionnaire, as independent variables in order to investigate the factors that were significantly associated with the risk of persistent paresthesia.

**Results:**

One hundred and seventy-six patients (6.44%) were found to have persistent paresthesia. Multivariate analysis revealed that surgical site at the extremities (odds ratio (OR), 12.5; 95% confidence interval (CI), 2.77–56.4; the reference was set at abdominal surgery), duration of general anesthesia (per 10 min) (OR, 1.02; 95% CI, 1.01–1.03), postoperative headache (OR, 1.78; 95% CI, 1.04–2.95), and days taken to visit the consultation clinic (OR, 1.03; 95% CI, 1.01–1.06) were independently associated with persistent paresthesia. Radiating pain was not significantly associated with persistent paresthesia (OR, 1.56; 95% CI, 0.69–3.54).

**Conclusion:**

Radiating pain during epidural procedure was not statistically significantly associated with persistent paresthesia, which may imply that this radiating pain worked as a warning of nerve injury.

## Introduction

Accidental puncture of the spinal cord or nerve roots elicits severe radiating pain in conscious patients. It has been suggested that radiating pain during spinal or epidural needle insertion and catheter placement can be an indicator of needle-related nerve injury [1]. Several studies have demonstrated that a neurologic deficit related to regional anesthesia techniques is most likely to develop in the same dermatome as the unusual pain or paresthesia that was elicited during needle insertion [2, 3]. Actually, in a review of the American Society of Anesthesiologists Closed Claims Project database, Cheney et al. reported that an associated risk factor of nerve root injuries associated with epidural anesthesia was paresthesia during needle/catheter placement [4]. Accordingly, radiating pain or paresthesia during epidural needle insertion and catheterization might be a sign of development of persistent nerve injury in some patients. On the other hand, there is an opinion that severe radiating pain is elicited in conscious patients, which at this time can be just a warning sign of nerve injury. Therefore, needle-related neurologic complications are likely to be avoided if the inserted needle is retracted promptly when the radiating pain is elicited [1]. According to this opinion, we usually provided epidural anesthesia in awake or lightly sedated status before providing general anesthesia. However, we may need to reconsider our practical attitude for epidural anesthesia if radiating pain per se during epidural needle insertion and catheterization could be strongly associated with persistent neurologic complications.

In our institute, epidural needle insertion and catheterization are performed in awake status according to this warning theory. To determine our practical attitude for epidural anesthesia, we need to investigate whether radiating pain or paresthesia during epidural anesthesia might be just a warning of nerve injury or a sign of development of persistent nerve injury. In this study, we used postoperative persistent paresthesia as an indicator for development of persistent nerve injury. Using a historical cohort, we investigated what factors could be associated with postoperative persistent paresthesia. In addition, we focused on radiating pain during epidural needle insertion and catheterization.

## Methods

We obtained approval for the review of patient clinical charts, for the access to data of the institutional registry of anesthesia, and for reporting of the results from the Institutional Review Board. The requirement for written informed consent was waived by the Institutional Review Board (No. 2724 approved on Sep-8-2020).

### Perioperative management

No standardization was conducted for the methods of anesthesia: epidural and general anesthesia. However, the methods of anesthesia did not significantly differ as this was a single-center study. No premedication was used. All patients had an epidural catheter inserted before general anesthetic induction. Epidural space was confirmed by using the loss-of-resistance technique combined with a median or paramedian approach in the left lateral position. The subcutaneous area at the targeted vertebral interspace was infiltrated with 1% lidocaine 3–5 mL. Subsequently, an 18-gauge Tuohy needle was carefully inserted into the epidural space. The inserted needle was retracted promptly, and the needle direction was changed when radiating pain was elicited. The epidural catheter was advanced 5 cm beyond the introducer needle tip. A test dose of 1% lidocaine 3 mL was administered to exclude unintentional subarachnoid injection. However, because confirmation of blockade area was time-consuming, there was no confirmation of a neural blockade by epidural injection of local anesthetics before the induction of general anesthesia. Postoperative analgesia was provided with epidural ropivacaine (0.1–0.2%, 2–4 mL/h) combined with fentanyl (10–20 μg/h) using a patient-controlled analgesia (PCA) device (Coopdech Balloonjector PCA Device^TM^, Daiken Medical Co. Ltd., Osaka City, Osaka, Japan). The PCA bolus size and lockout timing were set at 3 mL and 30 min, respectively. When PCA was used, a low-dose droperidol (1.25–2.5 mg/day) was combined with a PCA device.

After the completion of anesthesia, the attendant in charge filled out the form for the institutional registry of anesthesia, which included the following information: the attendant’s name, patient’s demographic variables, background illnesses, duration of anesthesia and surgery, American Society of Anesthesiologists physical status, urgency of surgery (emergency or elective), anesthesia technique (inhalational or intravenous with or without regional analgesia), surgical site, intraoperative patient positioning, requirement of transfusion, requirement of postoperative intensive care, intraoperative adverse intraoperative events, and use of a pneumatic compression device. As a general institutional rule, the patients visited the postoperative anesthesia consultation clinic after hospital discharge and completed a questionnaire using a self-report form. The questionnaire included items on intensity of postoperative pain (none = 0, a little = 1, painful = 2), headache, and persistent paresthesia; intensity of subcutaneous injection pain, which was pain with local infiltration anesthesia before an epidural procedure (none = 0, a little = 1, painful = 2); and experience of radiating pain throughout an epidural procedure. We also recorded how many days had passed since the operative day when the patient visited the postoperative anesthesia consultation clinic. Regarding persistent paresthesia, we asked patients a simple question like “Do you have any uncomfortable feeling at your legs? For example, numbness, tingling, pricking, chilling, or burning.” so that patients could easily understand the question.

### Data handling

**Fig. 1:**
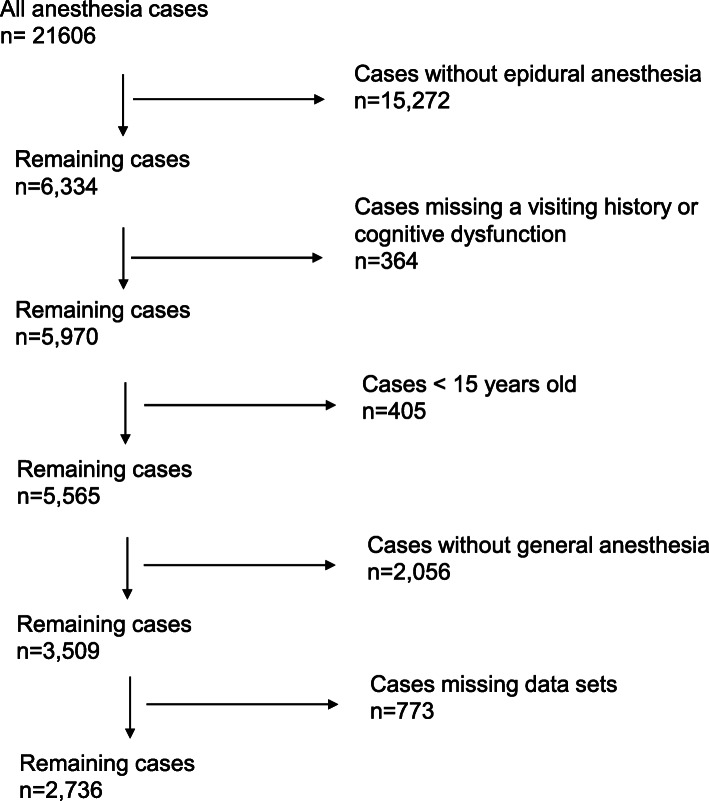
Exclusion criteria for the study

Data were collected between January 2009 and December 2013, during which period there were 21,606 anesthesia cases. The exclusion criteria for the current study (and the reasons for consequent reductions in eligible patients) were as follows: (1) cases without epidural anesthesia (*n* = 15,272), (2) cases missing a history of visiting the anesthesia consultation clinic or those who were unable to answer the questionnaire due to cognitive dysfunction (*n* = 364), (3) cases < 15 years old (*n* = 405), (4) cases without general anesthesia (*n* = 2056), and (5) cases missing anesthesia registry data sets or answers on the postoperative questionnaire (*n* = 773) (Fig. 1).

### Statistical analysis

Continuous variables are presented as mean and standard deviation if normally distributed or median and interquartile range if nonparametric. Categorical variables are presented as the number of patients. In the study cohort (2736 patients), we used univariate analysis to identify factors associated with postoperative persistent paresthesia. We conducted multivariate logistic analysis in the derivation cohort using the incidence of persistent paresthesia as a dependent variable and other covariates, including items of the anesthesia registry and the postoperative questionnaire, as independent variables in order to investigate the factors that were significantly associated with the risk of persistent paresthesia. We used candidate factors with a significant univariate association (*p* < 0.2) with persistent paresthesia to perform multivariable logistic regression analysis by forced-entry methods. We entered all candidate variables in the initial model and present them as adjusted odds ratios (ORs) with 95% confidence intervals (CI). We systematically searched the interactions between variables and considered collinearity for *r* or rho > 0.8 using Pearson or Spearman coefficient matrix correlation, respectively. We assessed the discrimination of the final model for dissatisfaction using the likelihood ratio test. We also calculated the area under the receiver operating characteristic curve to assess the performance of the model. We tested the calibration of the model using the Hosmer–Lemeshow statistic. Analyses were computed using the MedCalc statistical package (version 18.11.6, MedCalc Software bvba, Ostend, Belgium). *p* < 0.05 was considered statistically significant.

## Results

We analyzed data from 2736 patients, of whom 176, representing 6.44% of the overall population, were found to have persistent paresthesia. We compared patient data and perioperative characteristics between patients in both categories (Table 1). Univariate analysis revealed that older age, presence of coexisting disease, surgical sites, subcutaneous injection pain, radiating pain, requirement of transfusion, intraoperative adverse intraoperative events, duration of anesthesia and surgery, intensity of postoperative pain, postoperative headache, and longer time taken to visit the postoperative anesthesia consultation clinic were candidates associated with persistent paresthesia for the next multivariate analysis. We observed collinearity between duration of anesthesia and surgery. Therefore, duration of surgery was not entered in the final model.

**Table 1:** Results of univariate analyses

	Paresthesia (*n*=176)	No paresthesia (*n*=2560)	*P* value
Age (year)	63 (14)	64 (13)	0.191*
Sex (M/F)	100/76	1571/989	0.232
Height (cm)	161 (8)	161 (9)	0.301
Weight (kg)	59 (11)	59 (11)	0.968
BMI (kg m^−2^)	22.7 (3.6)	22.5 (3.4)	0.447
ASA physical status (1–5)	2 (2–2)	2 (2–2)	0.946
Coexisting disease (*Y*/*N*)	119/57	1876/686	0.114*
Resident management (*Y*/*N*)	103/73	1506/1054	0.937
Intensity of injection pain (0–2)	0 (0–1)	0 (0–0)	0.081*
Radiating pain (*Y*/*N*)	8/168	57/2503	0.067*
Duration of anesthesia (min)	376 (217)	324 (154)	0.00002*
Duration of surgery (min)	306 (213)	255 (148)	0.00002
Inhalational anesthesia (*Y*/*N*)	157/19	2261/299	0.808
Pneumatic compression (*Y*/*N*)	152/24	2254/306	0.475
Emergency (*Y*/*N*)	6/170	84/2476	0.828
Transfusion	51/125	495/2065	0.003*
Surgical posture (supine/lateral/lithotomy/other)	93/61/20/2	1413/893/223/31	0.652
Surgical site (abdominal/extremity/thoracic)	120/3/53	1822/5/733	0.0152*
Intraoperative adverse event (*Y*/*N*)	2/174	10/2550	0.178*
ICU admission	68/108	880/1680	0.253
Intensity of postoperative pain (0–2)	1 (1–2)	1 (0–1)	0.027*
Postoperative headache (*Y*/*N*)	179/2381	21/155	0.023*
Days taken to visit postoperative anesthesia consulting clinic	11 (7)	10 (5)	0.003*

Variables are expressed as number of patients, mean (SD), or median (IQR)

*Variables marked with an asterisk were entered into the logistic regression model

*BMI* body mass index, *ASA* American Society of Anesthesiologists, *ICU* intensive care unit

Multivariate analysis revealed that surgical site at the extremities (OR, 12.5; 95% CI, 2.77–56.4; the reference was set at abdominal surgery), duration of general anesthesia (per 10 min) (OR, 1.02; 95% CI, 1.01–1.03), postoperative headache (OR, 1.78; 95% CI, 1.04–2.95), and days taken to visit the consultation clinic (OR, 1.03; 95% CI, 1.01–1.06) were independently associated with persistent paresthesia (Table 2). Radiating pain was not significantly associated with persistent paresthesia (OR, 1.56; 95% CI, 0.69–3.54). Discrimination of the final models, assessed by the likelihood ratio test, was significant for these variables (*p* < 0.001). Hosmer–Lemeshow analysis suggested an acceptable calibration (*p* = 0.132). The forced-entry model had an area under the receiver operating characteristic curve of 0.648 (95% CI, 0.630–0.666).

**Table 2:** Results of multivariate analysis

Variables	Odds ratio	95% CI	*p* value
Age	1.00	0.98–1.01	0.557
Coexisting disease	1.31	0.92–1.89	0.137
Radiating pain	1.56	0.69–3.54	0.288
Intensity of injection pain	1.19	0.90–1.58	0.228
Duration of anesthesia (per 10 min)	1.02	1.01–1.03	0.0007
Surgical site			
Abdominal	1	NA	NA
Extremity	12.5	2.77–56.4	0.001
Thoracic	1.34	0.93–1.01	0.118
Transfusion	1.43	0.95–2.16	0.083
Adverse event	1.88	0.37–9.61	0.449
Intensity of postoperative pain	1.21	0.97–1.51	0.098
Postoperative headache	1.78	1.08–2.95	0.024
Days taken to visit postoperative anesthesia clinic	1.03	10.1–1.06	0.008

*NA* not applicable

A post hoc power calculation was conducted for this forced-entry multivariable logistic regression model using 11 variables. We followed standard methods to estimate the sample size for multivariable logistic regression, with at least 10 outcomes required for each included independent variable [5]. With an incidence of persistent paresthesia of 176/2734 (6.44%) in this population, we required 1708 patients to perform accurate multivariable logistic regression with 11 variables, which demonstrates that our sample size was sufficient to build the model.

## Discussion

This study demonstrated that radiating pain during an epidural procedure was not statistically significantly associated with persistent paresthesia. While at the same time, we found that cases with surgical site at the extremities, with longer duration of anesthesia, with postoperative headache, and who took longer to visit the postoperative anesthesia consultation clinic were all prone to have postoperative persistent paresthesia.

Radiating pain during an epidural procedure was not statistically significantly associated with persistent paresthesia, which may imply that this radiating pain was just a warning of nerve injury not a sign of neuronal injury. However, we must remember that this result was based on the theory that needle-related neurologic complications are likely to be avoided if the inserted needle is retracted promptly when the radiating pain is elicited [1]. Patients who canceled epidural anesthesia due to radiating pain were excluded from the study. In addition, we did not know exactly how many patients canceled epidural anesthesia for this reason. Such cases should not be ignored, though they have been very infrequent. The reason why we did was because it was difficult to extract them as cases of general anesthesia combined with epidural anesthesia. They were treated as cases of general anesthesia without epidural anesthesia from the viewpoint of our anesthesia registration. Therefore, it is still unknown whether severe radiating pain causing cancelation of epidural anesthesia could result in persistent paresthesia. In the study population included in our final analysis, however, we can say that radiating pain during an epidural procedure was not strongly associated with persistent paresthesia.

Some authors have suggested that the risk of neurologic complications associated with epidural catheter placement or spinal drain placement in anesthetized patients is small [6, 7]. However, considering that an isolated radiating pain unlikely causes permanent neurologic complication if the rule of promptly retracting the inserted needle by this alert is strictly respected, it is more acceptable that epidural procedure should be performed in awake status, as much as possible.

By the way, we had not provided cervical epidural anesthesia to any patients with a surgical site at the extremities. Thus, patients with upper extremity surgeries were excluded in this analysis. Therefore, it follows that lower extremity surgeries per se were associated with persistent paresthesia. This is just right because patients usually complain about paresthesia at surgical sites for a while. The postoperative questionnaire simply asked “Do you have any uncomfortable feeling at your legs?” This may be why patients’ response to the question was straight. In addition, the use of pneumatic tourniquet during orthopedic surgery could have influenced the incidence. Furthermore, postoperative longer fixation may be a plausible explanation in some cases.

Longer duration of anesthesia was also associated with persistent paresthesia. It has been suggested that the majority of intraoperative nerve injuries are associated with intraoperative positioning [8]. Therefore, it is reasonable that the longer the duration of anesthesia is, the more frequently intraoperative nerve injury can occur.

Headache was significantly associated with postoperative paresthesia. Headache treated in this study was not restricted to post-dural puncture headache (PDPH). Therefore, it does not directly mean that PDPH was closely associated with persistent paresthesia induced by nerve injury during epidural procedure. It has been reported that several patients experience postoperative depression [9, 10]. Population-based studies and clinical investigations found high rates of comorbidity between headache or paresthesia and depressive status having the characteristics of mood and anxiety disorders [11, 12]. These might have resulted in the present finding.

The number of days taken to visit the postoperative anesthesia consultation clinic was also associated with persistent paresthesia. It is obvious that patients with persistent paresthesia were sicker than patients without paresthesia. Therefore, it is supposed that it took more time for sicker patients to recover to the status that they could visit a post-anesthesia consultation by themselves. Otherwise, longer bed rest may have caused the longer nerve compression time, and it may have worsened the degree of nerve injury.

The current study had several limitations that merit discussion. First, this study was retrospective in nature; thus, unmeasured variables could still confound the results. We used data from the institutional registry of anesthesia, which includes minimal essential information about each case, but does not include precise details. Therefore, we did not obtain several variables which might have affected postoperative persistent paresthesia. For example, it was reported that multiple needle-insertion attempts were risk factors for neurologic deficits after epidural anesthesia [13]. However, the current study did not include information on the number of attempts. Second, this study relied on patient self-reports to determine symptoms. Therefore, postoperative paresthesia may include paresthesia induced by neural anesthesia, surgical tissue damage, or unsatisfactory patient positioning [13]. Therefore, it may be difficult to distinguish these etiologies. Third, a considerable number of patients were excluded from the study. However, the excluded patients might not have affected the results because the exclusion was performed according to the objective criteria, and the missing data were at least missing at random. Fourth, there should have been some deviations from our institutional anesthesia protocol because the methods of anesthesia were basically left to the preference of the anesthesia attendant. However, our hospital is a teaching hospital. Therefore, it is reasonable to think that the deviation from the standard protocol was not so large even though there were some deviations. Finally, the present study represents an audit of clinical practice at an individual institution, and our findings might not be generalizable to the practice of anesthesiology as a whole.

## Conclusion

Radiating pain during an epidural procedure was not statistically significantly associated with persistent paresthesia, which may imply that this radiating pain worked as a warning sign of nerve injury. However, we should remember that the rule of promptly retracting the inserted needle when the radiating pain is elicited may contribute to this result.

## Data Availability

They are available as a spreadsheet file. However, we would like to deal with individually.

## Abbreviations

PCA: Patient-controlled analgesia
ORs: Odds ratios
CI: Confidence intervals
